# Supplementary material for: Tree diet: reducing the treewidth to unlock FPT algorithms in RNA bioinformatics
Source: Algorithms Mol Biol. 2022 Apr 2;17:8. doi: 10.1186/s13015-022-00213-z (PMC8976393; doi:10.1186/s13015-022-00213-z)
Supplement: Supplementary file 1 — Additional file 1. Supplementary sections: A Editing Trees before the Diet; B Pseudo-code;C Correctness of the rejection-based sampling of RNA designs;D Lower bound for the min. alignment cost from simplified models. [file 13015_2022_213_MOESM1_ESM.pdf]

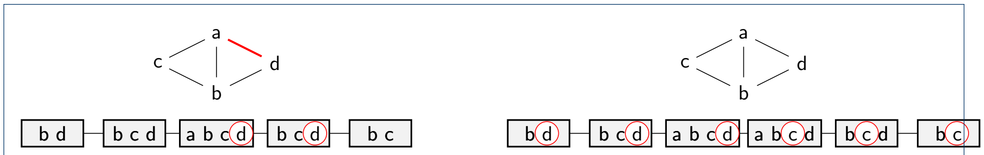

Figure 12 Left: A graph and a path-decomposition whose optimal 1-tree-diet loses an edge (ad). However, duplicating the bag abcd (right) yields a tree-decomposition with a lossless 1-tree-diet.

Supplementary Material
Section A: Editing Trees before the Diet

Any tree decomposition can be transformed into a binary one through the duplications of bags having more than 2 children. To do so in practice, one will, as long as the tree decomposition is not binary, apply the following transformation:

1. Find a bag X with children $Y_1, \ldots, Y_\Delta$ and $\Delta > 2$.
2. Introduce a new bag X' with the same content as X and locally modify the tree decomposition in the following way: X will now have Y_1 and X' as children, while X' will have $Y_2 \cdots Y_\Delta$.

When it is no longer possible to apply this transformation, the tree decomposition is binary. For each bag having originally $\Delta > 2$ children in the decomposition, $\Delta - 1$ new bags have been introduced. In total, with N_{bags} the original number of bags in the decomposition, strictly less than N_{bags} new bags have been introduced (each new bag is associated to an edge of the original tree decomposition).

This tranformation is in fact the first step towards obtaining a *nice tree decomposition* [42, 29].

A question that arises then is what impact these modifications may have on the output of Tree-Diet, when applied to the tree decomposition given as input. We argue that duplication operations (as used above to get a binary tree decomposition) can only improve the solution, i.e decrease the number of *lost* edges. Indeed, within the coloring formulation of the problem, new bags yield new opportunities for an edge to be *represented*, with both its end-points green in some bag. See Figure 12 for an illustration.

More generally, any operation on the input tree decomposition that does not suppress any of the original bags can only improve the solution to the Tree Diet problem. We do not tackle here the problem of finding the best edition operations to apply onto a tree decomposition given as input to Tree Diet, which is an a priori difficult task.

Section B: Pseudo-code

Algorithm 1 and 2 present a pseudo-code of our dynamic programming algorithm for Tree Diet, with a memoization approach. The C++/pybind11 [44] implementation is available at https://gitlab.inria.fr/amibio/tree-diet.

Note that the implementation allows to solve a more general *weighted* version of Tree Diet, where each edge is given a weight, and the objective is to find a $(tw-tw')$-diet of the input tree decomposition preserving a set of edges of maximum total weight.

In the context of RNA applications, this feature allows to favour as much as possible preservation of the backbone of RNA molecules, i.e. edges between consecutive

nucleotides along the string, by assigning them a weight greater than the number of non-backbone edges.

Edge weights are passed to the function in the form of a dictionary/map W associating a real weight to each edge. Within Algorithm 1, the only place where it is taken into account is the the *count* function, which computes the weight of edges accounted for by the bag that is currently visited.

Input : Tree-decomposition $\mathcal{T}$, graph G, target width tw', edge weights W
Output : Maximum total weight of a set of realizable/non-lost edges in a $(tw - tw')$-diet of $\mathcal{T}$
Side-Product: A filled table $c[X_i, f]$, $\forall X_i$ bag and f coloring of X_i

Function `optim_num_real_edges`(X_i, f, G, tw', W):
  if $c[X_i, f]$ *already computed* **then** return $c[X_i, f]$; ;
  if $|f^{-1}(o) \cup f^{-1}(r)| \leq (|X_i| - tw' - 1)$ **then**
    //not enough removals.;
    $c[X_i, f] = -\infty$;
    return $c[X_i, f]$;
  end
  if $X_i == leaf$ **then**
    $c[X_i, f] = 0$;
    return $c[X_i, f]$;
  end
  int ans $= -\infty$;
  for $m \in orange\_maps(X_i, f)$ **do**
    int ans_m $= 0$;
    for $Y_j \in X_i.children$ **do**
      int ans_j $= -\infty$;
      for $f'_j \in compatible(f, m, X_i, Y_j)$ **do**
        int val $= 0$;
        val $+=$ $count(f, f'_j, W)$;
        val $+=$ `optim_num_real_edges`(Y_j, f'_j, G, tw');
        if $val \geq ans\_j$ **then** ans_j $=$ val;
        ;
      end
      ans_m $+=$ ans_j;
    end
    if $ans\_m \geq ans$ **then** ans $=$ ans_m; ;
  end
  $c[X_i, f] = ans$
  return $c[X_i, f]$;
end

Algorithm 1: Dynamic programming algorithm for Tree-Diet.

Section C: Correctness of the rejection-based sampling of RNA designs

A recent method for RNA design, called RNAPond [22], implements a sampling approach to tackle the inverse folding of RNA. Targeting a secondary structure S of length n, it performs a Boltzmann-weighted sampling of sequences and, at each iteration, identifies Disruptive Base Pairs (DBPs) that are not in S, yet are recurrent in the Boltzmann ensemble of generated sequences. Those base pairs are then added to a set $\mathcal{D}$ of DBPs, and excluded in subsequent generations through an assignment of non-binding pairs of nucleotides, outside of $\mathcal{B} := \{(\mathsf{G}, \mathsf{C}), (\mathsf{C}, \mathsf{G}), (\mathsf{A}, \mathsf{U}), (\mathsf{U}, \mathsf{A}), (\mathsf{G}, \mathsf{U}), (\mathsf{U}, \mathsf{G})\}$.

```
Input  : Tree-decomposition 𝒯, graph G, target width tw', table c, edge weights W
Output : Optimal (tw − tw')-diet-valid coloration for 𝒯
Function optim_coloring(X_i, f, G, tw', c):
    if X_i == leaf then
        return ∅;
    end
    coloring 𝒞 = ∅;
    for m ∈ orange_maps(X_i, f) do
        int ans_m = 0;
        coloring best_fjs = [];
        for Y_j ∈ X_i.children do
            int best_valj = −∞;
            int best_fj = ∅;
            for f'_j ∈ compatible(f, m, X_i, Y_j) do
                int val = 0;
                val += count(f, f'_j, W);
                val += c[Y_j, f'_j];
                if val ≥ best_valj then
                    best_valj = val;
                    best_fj = f'_j;
                end
            end
            ans_m += best_valj;
            best_fjs.add(best_fj);
        end
        if ans_m == c[X_i, f] then
            𝒞+ = [f'_j for f'_j in best_fjs];
            𝒞+ = [optim_coloring(Y_j, f'_j, G, tw', c) for f'_j in best_fjs];
            break;                                        // break loop over m
        end
    end
    return 𝒞;
end
```

Algorithm 2: Backtracking procedure for TREE-DIET.

At the core of the method, one finds a random generation algorithm which takes as input a secondary structure S and a set $\mathcal{D}$ of DBPs. The algorithm generates from the set $\mathcal{W}_{S,\mathcal{D}}$ of sequences $w \in \{\mathsf{A}, \mathsf{C}, \mathsf{G}, \mathsf{U}\}^n$ which are: i) compatible with all $(i,j) \in S$, *i.e.* $(w_i, w_j) \in \mathcal{B}$; and ii) incompatible with all $(k,l) \in \mathcal{D}$, *i.e.* $(w_k, w_l) \notin \mathcal{B}$. The algorithm then enforces a (dual) Boltzmann distribution over the sequences in $\mathcal{W}_{S,\mathcal{D}}$:

$$\forall w \in \mathcal{W}_{S,\mathcal{D}} : \mathbb{P}(w \mid \mathcal{D}, S) = \frac{e^{-\beta.E_{w,S}}}{\mathcal{Z}_{S,\mathcal{D}}} \quad \text{with} \quad \mathcal{Z}_{S,\mathcal{D}} := \sum_{w' \in \mathcal{W}_{S,\mathcal{D}}} e^{-\beta.E_{w',S}} \quad (1)$$

where $\beta > 0$ is an arbitrary constant akin to a temperature. Yao *et al.* describe an algorithm which generates k sequences in $\Theta(k(n+|\mathcal{D}|))$ time, after a preprocessing in $\Theta(n.|\mathcal{D}|.4^{tw})$ time and $\Theta(n.4^{tw})$ space, where tw is the treewidth of the graph having edges in $S \cup \mathcal{D}$.

The discrepancy in the preprocessing and sampling complexities suggests an alternative strategy, utilizing rejection on top of a relaxed sampling. Namely, we consider a rejection algorithm, which starts from a relaxation $(S', \mathcal{D}')$ of the initial constraints $(S' \cup \mathcal{D}' \subset S \cup \mathcal{D})$, and iterates Yao *et al.*'s algorithm to generate sequences in $\mathcal{W}_{S',\mathcal{D}'} \supset \mathcal{W}_{S,\mathcal{D}}$, rejecting those outside of $\mathcal{W}_{S,\mathcal{D}}$, until k suitable ones are obtained. The rejection algorithm generates a given sequence $w \in \mathcal{W}_{S,\mathcal{D}}$ on its first attempt with probability $p := e^{-\beta.E_{w,S}}/\mathcal{Z}_{S',\mathcal{D}'}$ and, more generally, after r rejections with probability $(1-q)^r\, p$ with $q := \mathcal{Z}_{S,\mathcal{D}}/\mathcal{Z}_{S',\mathcal{D}'}$. The overall probability of emitting w is thus

$$p \cdot \sum_{r \geq 0} (1-q)^r = \frac{p}{q} = \frac{e^{-\beta.E_{w,S}}}{\mathcal{Z}_{S,\mathcal{D}}} = \mathbb{P}(w \mid \mathcal{D}, S).$$

In other words, our relaxed generator coupled with the rejection step, represents an unbiased algorithm for the Boltzmann distribution of Eq. (1) over $\mathcal{W}_{S,\mathcal{D}}$.

Meanwhile, the average-case complexity can be impacted by the strategy. Indeed, the relaxed instance $(S', \mathcal{D}')$ can accelerate the preprocessing due to a reduced treewidth $tw' \leq tw$. The rejection step only increases the expected number of generations by a factor $\overline{q} := \mathcal{Z}_{S',\mathcal{D}'}/\mathcal{Z}_{S,\mathcal{D}}$, representing the inflation of the sequence space, induced by the relaxation of the constraints. Overall, the average-case time complexity of the rejection algorithm is in $\Theta(n \cdot |\mathcal{D}'| \cdot 4^{tw'} + k \cdot \overline{q} \cdot (n + |\mathcal{D}'|))$ time and $\Theta(n \cdot 4^{tw'})$ space. This space improvement is notable when $tw' < tw$, and could be key for the practical applicability of the method, especially given that memory represents the bottleneck of most treewidth-based DP algorithms.

Section D: Lower bound for the min. alignment cost from simplified models

Here, we justify the filtering strategy described in Section 5.2.2. Namely, we formally prove that, given a structured RNA S and a targeted genomic region w, a lower bound for the minimal alignment cost of S and w can be obtained from the minimal alignment cost of some $S' \subseteq S$ and w. If this lower bound for $S' \subseteq S$ is higher than the specified cutoff ε, then there is no need to align w to the full model S, as the resulting cost is guaranteed to stay above the selection cutoff ε.

Let S be an arc-annotated sequence of length m (S_i denotes the ith character of S), w be a target (flat) sequence of length m, and $\mu : [1,n] \rightarrow [1,m] \cup \{\perp\}$ represents an alignment[1]. We consider the following cost function, adapted from eciteRinaudo2012, which quantifies the quality of an alignment μ for S and w:

$$C(S,w,\mu) = \sum_{\substack{i \text{ unpaired in } S,\\ k:=\mu_i}} \gamma(S_i, w_k) + \sum_{\substack{(i,j)\in S,\\ (k,l):=(\mu_i,\mu_j)}} \phi(S_i,S_j,w_k,w_l) + \sum_{g\in gaps(S)} \lambda_q(g) + \sum_{g\in gaps(w)} \lambda_T(g)$$

where

- $\gamma(a,b)$ returns the *substitution cost* which penalizes (mismatches) or rewards (matches) the substitution of a into b (set to 0 and handled in gaps if $b = \perp$);
- $\phi(a,b,c,d)$ returns a *base pair substitution cost*, penalizing (arc breaking) or rewarding (conservation or compensatory mutations) the transformation of nucleotides (a,b) into nucleotides/gaps (c,d) (set to 0 and handled in gaps if $(c,d) = (\perp,\perp)$);
- λ_S and λ_T penalize gaps introduced by μ respectively in S and w (affine cost model).

Given this definition, consider a simplified model $S' \subset S$, associated with a minimal cost

$$c' := \min_{\mu} C(S,w,\mu)$$

and denote by $c^\star$ the minimal cost of the full model S, we have the following inequality.

Proposition 3

$$c' - \sum_{\substack{i \text{ unpaired in } S,'\\ \text{paired in } S}} \max_b \gamma(S_i,b) + \sum_{(i,j)\in S\setminus S'} \min_{a,b} \phi(S_i,S_j,a,b) \leq c^\star \qquad (2)$$

Proof For any alignment, we have, per the definition of $C(S,w,\mu)$:

$$C(S,w,\mu) = C(S',w,\mu) - \sum_{\substack{i \text{ unpaired in } S,'\\ \text{paired in } S,\\ \text{and } k:=\mu_i}} \gamma(S_i,w_k) + \sum_{\substack{(i,j)\in S\setminus S'\\ \text{s.t. } (k,l):=(\mu_i,\mu_j)}} \phi(S_i,S_j,w_k,w_l).$$

Minimizing over all alignment μ, one obtains

$$\min_{\mu} C(S,w,\mu) = \min_{\mu} C(S',w,\mu) - \sum_{\substack{i \text{ unpaired in } S,'\\ \text{paired in } S,\\ \text{and } k:=\mu_i}} \gamma(S_i,w_k) + \sum_{\substack{(i,j)\in S\setminus S'\\ \text{s.t. } (k,l):=(\mu_i,\mu_j)}} \phi(S_i,S_j,w_k,w_l).$$

[1] An alignment μ is subject to further constraints, notably including some restricted form of monotonicity, when represented as a function. However, those constraints are reasonably intuitive and we omit them in this discussion for the sake of simplicity.

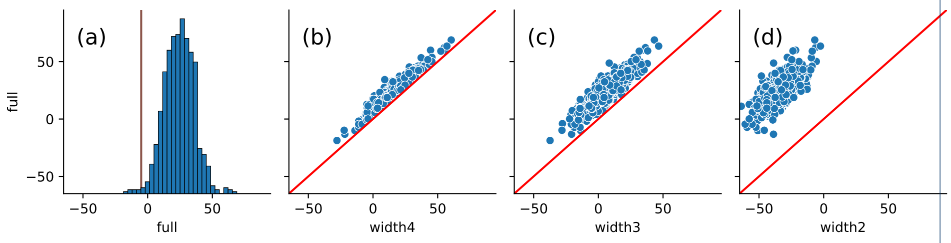

Figure 13 (a) Histogram of alignment scores obtained by aligning the full structure ($tw = 5$) model of the Twister ribozyme (pdb-id: 4OJI) with $\kappa \cdot n$-sized windows in a 10kb region of the 5th chromosome of *S. bicolor*. A vertical line is positioned at the ϵ threshold. (b;c;d) Corrected alignment scores obtained for reduced-treewidth models for each window, plotted against the corresponding score of the full model. The corrected alignment score indeed acts as a lower bound to the full-model score (points above the $y = x$ red line), allowing an iterative filtering strategy.

Independently minimizing each term of the right-hand-side, we obtain a first lower bound

$$c^\star \geq c' - \max_{\mu} \sum_{\substack{i \text{ unpaired in } S,' \\ \text{paired in } S, \\ \text{and } k := \mu_i}} \gamma(S_i, w_k) + \min_{\mu} \sum_{\substack{(i,j) \in S \setminus S' \\ \text{s.t. } (k,l) := (\mu_i, \mu_j)}} \phi(S_i, S_j, w_k, w_l).$$

further coarsened by an independent optimization of the elements in the sums

$$\begin{aligned} c^\star &\geq c' - \sum_{\substack{i \text{ unpaired in } S,' \\ \text{paired in } S}} \max_{\mu} \gamma(S_i, w_k) + \sum_{(i,j) \in S \setminus S'} \min_{\mu} \phi(S_i, S_j, w_k, w_l) \\ &= c' - \sum_{\substack{i \text{ unpaired in } S,' \\ \text{paired in } S}} \max_{a} \gamma(S_i, a) + \sum_{(i,j) \in S \setminus S'} \min_{a,b} \phi(S_i, S_j, a, b). \end{aligned}$$

where the last line is obtained by considering the worst-case contributors to nucleotides and base pairs substitutions. Importantly, the right-hand side no longer depends on μ any more, and can be used to easily computed a corrected score/lower bound. □

The corrected expression, shown in the left hand side of Equation (2) allows, when lower than a cutoff ε, to safely discard w as a potential hit for the full model S. This corrected score is plotted in Figure 11A, allowing for a gradual reduction of the search space for ε-admissible hits. We show in Figure 13 the corrected scores obtained for simplified structures S' of various treewidths, plotted against the scores of the full target structure.
